# Characteristics of Bicycle-Related Maxillofacial Injuries Between 2019–2023—Retrospective Study from Poznan, Poland

**DOI:** 10.3390/jcm14176075

**Published:** 2025-08-28

**Authors:** Kacper Nijakowski, Szymon Rzepczyk, Maria Szczepaniak, Jakub Majewski, Jakub Jankowski, Czesław Żaba, Maciej Okła

**Affiliations:** 1 Department of Conservative Dentistry and Endodontics, Poznan University of Medical Sciences, 60-812 Poznan, Poland; 2 Department of Forensic Medicine, Poznan University of Medical Sciences, 60-806 Poznan, Poland; 3 The Student Scientific Society of Poznan University of Medical Sciences, Poznan University of Medical Sciences, 60-806 Poznan, Poland; majek.sz@gmail.com (M.S.);; 4 Department of Maxillofacial Surgery, Poznan University of Medical Sciences, 60-355 Poznan, Poland; maciejokla@ump.edu.pl

**Keywords:** bicycle accidents, facial trauma, maxillofacial injury, facial fracture, road safety

## Abstract

**Background**: Bicycles constitute a primary means of transportation, particularly within the scope of urban micromobility. However, the use of this mode of transport is associated with the risk of traffic accidents and subsequent maxillofacial trauma. Cyclists are classified as vulnerable road users, among whom the assessment of injury patterns is a significant issue. This study aimed to identify the most common maxillofacial fractures resulting from bicycle-related traffic accidents. **Methods**: A retrospective analysis was conducted on the medical records of patients treated at the Clinic of Maxillofacial Surgery at the University Clinical Hospital in Poznan, who sustained maxillofacial injuries as a result of bicycle-related accidents between 2019 and 2023. **Results**: A total of 99 patients met the inclusion criteria. Most of the study population was males (70.7%), with a median age of 38. Accidents most frequently occurred during the summer months and on Fridays and weekends. The most common fracture site was the mandible (40.4%), with double fractures being the predominant type. Additionally, zygomatic-orbital fractures were frequently observed (30.3%). In terms of treatment, surgical intervention was predominant, and the mean duration of hospitalisation was 6 days. Only 5.1% of patients were under the influence of alcohol at the time of the incident. Furthermore, it was found that isolated mandibular fractures occurred more frequently in younger patients, whereas midface fractures of the Le Fort II and III types were more commonly observed in individuals under the influence of alcohol at the time of the event. Moreover, accidents involving alcohol consumption were associated with a higher incidence of concomitant cranio-cerebral injuries. **Conclusions**: Defining the profile of maxillofacial fractures resulting from bicycle accidents constitutes a clinically relevant issue. Additionally, identifying the main risk factors and developing preventive measures is of critical importance.

## 1. Introduction

Issues related to the safety of road users are of significant concern from both social and medical perspectives. This is particularly relevant for unprotected road users like cyclists [1,2,3]. Bicycles have become one of the key modes of transport utilised by the public, especially in urban environments. In many European cities, bicycles constitute an essential component of urban micromobility, forming a crucial link in the transportation chain by enabling users to bypass traffic congestion and navigate dense urban areas efficiently [4,5,6,7].

As a result, bicycles are commonly used for commuting to work or educational institutions, attending social events, shopping, and participating in cultural activities. Other factors contributing to the popularity of this mode of transportation include its eco-friendliness, zero emissions, low purchase and maintenance costs, and associated health benefits. Consequently, bicycles are also widely used for sports and recreational purposes, becoming a lifestyle element for many individuals [8]. The introduction of short-term bike rental systems in urban areas has further contributed to the growing interest in this form of transport [9,10].

The widespread use of bicycles and the increasing number of cyclists are associated with a heightened risk of traffic accidents [11,12]. Analysis of police statistics on bicycle-related accidents in Poland between 2016 and 2023 indicates that, on average, over 3500 such incidents occur annually, resulting in more than 3200 injuries and over 100 fatalities yearly [13]. Moreover, accidents are more frequently reported in urban areas. These incidents may result from collisions with other vehicles (e.g., passenger cars), impacts with obstacles (e.g., road safety barriers), or single-vehicle falls [14,15,16]. Contributing factors also include the quality of infrastructure, driving culture, compliance with traffic regulations, travelling speed, weather conditions, and lighting [17,18,19].

Such factors result in injuries requiring detailed analysis for developing emergency response protocols in hospital emergency departments and for event reconstruction in legal investigations carried out by relevant authorities. The head is one of the regions particularly susceptible to injury [15,20,21,22,23]. Furthermore, the face houses the entry points of the digestive and respiratory systems as well as critical sensory organs, making it especially vulnerable to injuries that can significantly affect the victim’s quality of life. Research indicates that one of the most common causes of maxillofacial trauma is traffic accidents, particularly those involving bicycles [24,25,26]. Previous reports estimated that craniofacial fractures may occur in 7% to 13% of all victims of bicycle-related accidents [27,28].

This retrospective study aimed to determine the typical profile of craniofacial injuries sustained by victims of bicycle-related accidents, along with an assessment of the circumstances accompanying these incidents.

## 2. Materials and Methods

A retrospective analysis was conducted on the medical records of patients treated at the Department and Clinic of Maxillofacial Surgery at the University Clinical Hospital of the Poznan University of Medical Sciences over a five-year period, from January 2019 to December 2023. The inclusion criterion for the study group was documentation indicating the occurrence of maxillofacial trauma as the result of a bicycle-related accident. Cases described in the medical records as “unspecified trauma” or “traffic accident” without further detail regarding the mechanism of injury were excluded from the analysis. Also, only conventional bicycles were considered during analysis; no electric bicycles were reported.

Eligible cases were examined in terms of demographic variables (gender and age), the blood presence of alcohol or psychoactive substance use at the time of the incident, temporal and situational circumstances of the accident, and the nature of sustained injuries, with a particular focus on anatomical location (each type of fracture was coded as 0–1 for each patient. Additionally, data on the length of hospitalisation, treatment modality, and the need for specialist consultations were evaluated. Two researchers collected all of the above data from the electronic medical record database into an Excel spreadsheet, which was then processed and subjected to statistical analysis.

This article represents a continuation of previous research concerning the characterisation of maxillofacial injuries concerning aetiology and contextual circumstances [29].

### Statistical Analysis

Qualitative variables were analysed using Pearson’s Chi-squared test, while quantitative variables were assessed with the Mann–Whitney U test, as the data did not follow a normal distribution according to the Shapiro–Wilk test. Continuous variables are reported as medians with interquartile ranges. A multidimensional correspondence analysis was conducted to examine the associations between the most common fracture types and selected patient characteristics (gender, age, and alcohol consumption). The three-dimensional solution was selected based on the scree plot, as it provided the most informative representation of the data. All statistical tests were two-tailed, with a significance threshold set at α = 0.05. Statistical analyses were performed using Statistica software, version 13.3 (StatSoft, Cracow, Poland).

## 3. Results

This retrospective analysis included 99 patients, comprising 29 females and 70 males. The median age was 38 years, and the median hospital stay lasted 6 days. The majority of individuals were of Polish nationality. Only 5.05% of patients tested positive for alcohol in their bloodstream. Table 1 provides detailed demographic information.

Table 2 outlines the distribution of injuries by year, month, and day of the week. The highest number of cases was recorded in 2020 and 2021, accounting for 58.58% of all injuries. June saw the peak in monthly cases (16.16%), and Fridays had the highest frequency of incidents, with 20.20% occurring that day.

Table 3 presents a comprehensive overview of the injuries. The mandible emerged as the most commonly injured area (44.44%). Among the fracture types, double fractures of the mandible were the most frequent (19.19%), closely followed by single mandible fractures (18.18%). The second most common type of injury was zygomaticomaxillary complex (ZMO) fractures (30.30%). In contrast, LeFort 3 fractures were the rarest, accounting for just 1.01% of cases. Fractures tended to affect both sides of the face in 42.42% of patients. In the majority of cases (70.70%), the cause of injury was not clearly defined. Additionally, other craniocerebral injuries were sustained by 5 of them.

Table 4 presents a comparative analysis of fracture types, injury mechanisms, and treatment modalities stratified by gender, age group (≤30 years vs. >30 years), and blood alcohol presence at admission. Mandibular fractures were observed across all subgroups, with significant differences in certain patterns. Double mandible fractures were significantly more prevalent among females (34.48%) compared to males (12.86%) (*p*-value = 0.013). Conversely, LeFort type II fractures occurred exclusively in male patients (12.86%) and were absent in females, reaching statistical significance (*p*-value = 0.043).

Age was also associated with fracture patterns. Single mandible fractures were significantly more common in younger patients (≤30 years), affecting 33.33% compared to 11.59% in older individuals (*p*-value = 0.010). Additionally, complex mandibular fractures involving three or more sites were significantly more prevalent in the younger cohort (16.67% vs. 2.90%, *p*-value = 0.014). Alcohol presence at the time of injury was strongly associated with certain fracture types. Frontal bone fractures were significantly more frequent among alcohol-positive patients (60%) compared to those without alcohol in their system (7.45%) (*p*-value < 0.001). LeFort type II (40%, *p*-value = 0.014) and type III fractures (20%, *p*-value < 0.001) were also significantly more common in the alcohol-positive group.

When comparing fracture combinations by gender, no statistically significant differences were observed. Mandible-only fractures were slightly more common in females than in males (44.83% vs. 32.86%, *p*-value = 0.519). Age-related analysis revealed a significant association with fracture type. Patients aged ≤30 years had a markedly higher rate of isolated mandible fractures compared with those >30 years (60.0% vs. 26.09%, *p*-value = 0.007). Conversely, non-mandible maxillofacial fractures were more frequent in the older group (single 43.48% vs. 23.33%, and multiple 23.19% vs. 6.67%). Alcohol consumption also showed a significant relationship with fracture patterns. Individuals with alcohol use had no isolated mandible fractures compared with 38.3% in non-users (*p*-value = 0.016). Instead, alcohol users more frequently presented with combined fractures, both with mandible involvement (40.0% vs. 6.38%) and without mandible involvement (40.0% vs. 17.02%), though the small number of alcohol-positive cases limits interpretation.

There were no statistically significant differences in fracture laterality (left, right, or bilateral) across gender, age, or alcohol status. However, bilateral fractures were notably more frequent among alcohol-positive individuals (80%) compared to alcohol-negative patients (40.43%), though this difference did not reach statistical significance (*p*-value = 0.189).

The aetiology of injury varied significantly by gender. A significantly higher proportion of male patients sustained injuries classified as undefined (80%) compared to females (48.28%) (*p*-value = 0.002). No statistically significant differences in injury cause were observed across age groups or based on alcohol consumption, although falls were more frequently reported among alcohol-positive patients (40%).

The vast majority of patients underwent surgical management. However, a significant difference was observed with respect to alcohol status: operative treatment was administered in 97.87% of alcohol-negative patients, compared to only 80% of alcohol-positive patients (*p*-value = 0.023). Ambulatory treatment was more common among alcohol-positive patients (20%), although not statistically tested for significance.

Alcohol-positive patients were significantly more likely to present with concomitant craniocerebral injuries (40% vs. 3.19%, *p*-value < 0.001). Furthermore, they more frequently required consultations with additional specialists (60% vs. 13.83%, *p*-value = 0.006).

Table 5 compares hospitalisation duration based on gender, age, and alcohol presence. Hospitalisation was longer for females compared to males, without statistical significance. Patients aged 30 or younger also required longer hospitalisation than those older than 30, although this difference was not statistically significant. Patients who tested positive for alcohol had a significantly longer hospitalisation, with a median of 11 days (*p*-value = 0.043).

**Figure 1:**
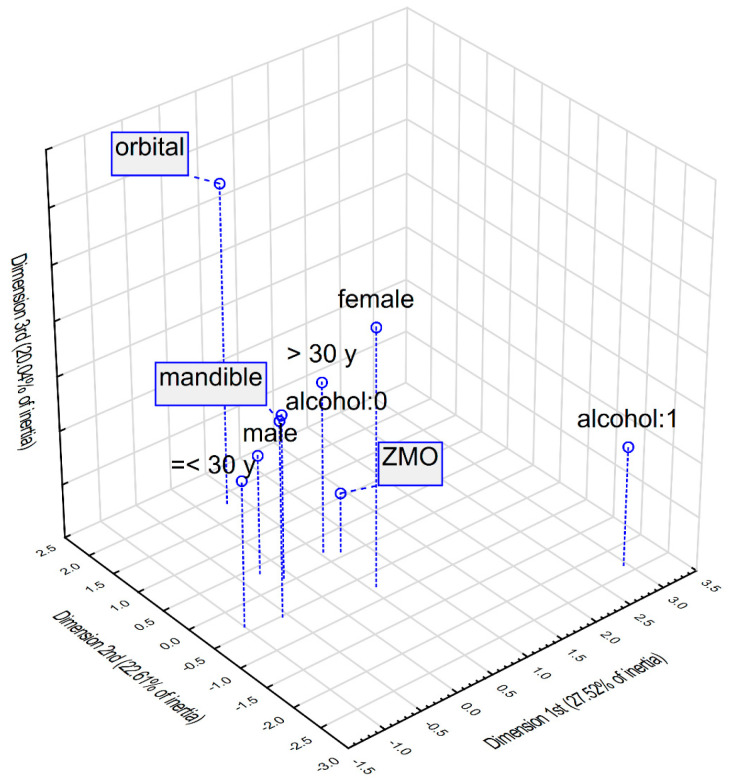
Multidimensional correspondence analysis—3-dimensional plot. Legend: “alcohol:0”—no alcohol consumption, “alcohol:1”—alcohol consumption. Interpretation: The plot displays the relationships between different categorical variables. Points that are located closer to one another suggest a stronger association, meaning they are more likely to occur together within the study population (e.g., “mandible” and “male”—males were more likely to sustain mandible fractures). In contrast, categories that are widely separated on the plot indicate little or no relationship (e.g., “orbital” and “alcohol:1”—orbital fractures were less commonly seen among alcohol drinkers).

**Figure 2:**
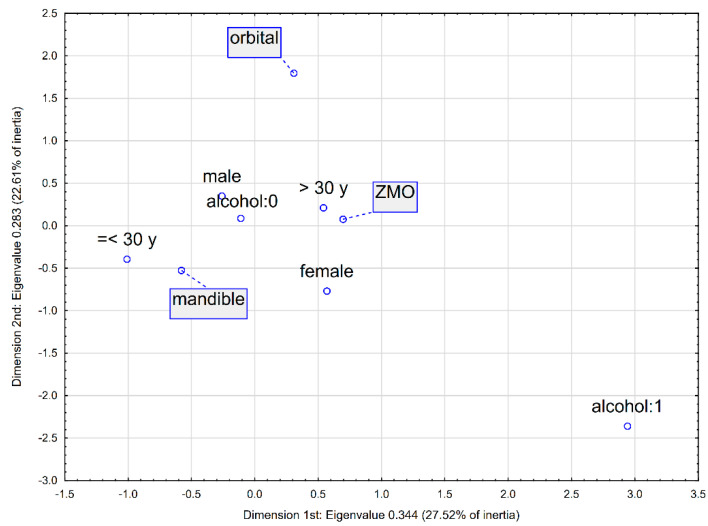
Multidimensional correspondence analysis—2-dimensional plot with the highest inertias. Legend: “alcohol:0”—no alcohol consumption, “alcohol:1”—alcohol consumption. Interpretation: In the case of two-dimensional analysis, the strength of the relationship is additionally determined by assessing the angle between the variables under study (with the vertex at the origin of the coordinate system, 0,0)—the smaller the angle, the stronger the relationship. For example, strong relationships are observed between age below 30 years and mandibular fractures, as well as between age above 30 years and ZMO fractures.

A multidimensional correspondence analysis was performed to explore the associations between the most common fracture types visually and selected patient variables, including gender, age, and alcohol consumption. The outcomes of this analysis are illustrated in Figure 1 and Figure 2, representing three-dimensional and two-dimensional plots, respectively, with the latter displaying the dimensions accounting for the highest inertia. Descriptive parameters for the plotted points are provided in Table 6.

The three-dimensional visualisation reveals that the points corresponding to male gender, age under 30, and absence of alcohol intake cluster most closely with mandible and orbital fractures, suggesting a stronger likelihood of these fracture types in this subgroup. The two-dimensional plot further supports this association, as it forms the smallest angle at the origin of the coordinate system. In contrast, the two-dimensional analysis also highlights a distinct association between ZMO fractures and patients who are female, over the age of 30, and have consumed alcohol.

## 4. Discussion

Traffic accidents involving cyclists most frequently occurred during the warm spring and summer months, with the highest weekly incidence observed on Fridays. Furthermore, males were 2.4 times more likely to be involved in such incidents, and the study population was predominantly young adults. Only 5% of the victims were under the influence of alcohol at the time of the bicycle-related accident.

Maxillofacial fractures were most commonly located in the mandible, often presenting bilaterally. Injury analysis by gender revealed that women were more likely to sustain double mandibular fractures, whereas men more frequently suffered from Le Fort II midfacial fractures. Younger patients were more likely to experience isolated mandibular fractures. Additionally, frontal bone fractures were more frequently observed in individuals who were under the influence of alcohol at the time of the incident.

The reported associations are unadjusted and may be influenced by confounding factors. It should be noted that a large proportion of injuries in this cohort were categorised under undefined causes. This constrains the ability to establish clear associations between specific aetiological factors and fracture patterns, and the findings regarding demographic and behavioural variables (such as age and alcohol consumption) should be interpreted with caution.

Analyses of bicycle-related accidents conducted in Poland indicate a general decline in such incidents in recent years [13]. However, in the present study cohort, a noticeable increase in accidents was observed in 2020 and 2021. This may be attributed to the growing interest in cycling during that period, partly driven by public transportation restrictions imposed due to the COVID-19 pandemic, specifically passenger limits per vehicle, which compelled many commuters to seek alternative modes of transport. Restrictions also affected venues for physical activity (e.g., gyms), prompting individuals to adopt cycling as an alternative form of exercise [30,31,32,33].

Previous research has shown that bicycle accidents resulting in injury are more likely to occur during warm summer [34]. This trend was also observed in the present study cohort of patients who sustained maxillofacial trauma. Additionally, weekend days showed a slightly higher frequency of bicycle-related injuries than weekdays [34]. Some studies suggest that these incidents are more likely to occur in the morning [35]. However, a study conducted in Japan reported a higher incidence of bicycle-related maxillofacial fractures on weekdays and during evening hours [36].

Analyses of traffic accidents involving cyclists indicate that the victims are most often men, who may account for over 80% of the injured overall [37,38]. Additionally, studies show that victims are usually adults under the age of 50, who may constitute up to half of all bicycle-related trauma patients admitted to trauma centres [39]. Similar observations were noted in the present study cohort of patients who sustained craniofacial injuries as a result of bicycle-related accidents.

Maxillofacial injuries resulting from bicycle accidents are typically caused by blunt trauma to the facial region. The energy of the trauma depends on the specific circumstances of the event, including the speed at the time of the accident. Analysis of bicycle-related maxillofacial injuries shows that fractures most commonly occur in the mandible [27,36,40,41]. This finding was mirrored in the present study, where mandibular fractures were observed in over 44% of cases. Mandibular fractures most often involve the condyle and symphysis [36,42], while fractures of the body and angle of the mandible are somewhat less frequent [40,42].

Some studies, however, identify the zygomaticomaxillary complex as the most common fracture site [43,44]. Furthermore, research in Italy indicates that the mandible is the most frequently fractured site in paediatric patients involved in bicycle-related accidents [45]. When evaluating midface fractures among victims of bicycle accidents, Le Fort I and Le Fort II fractures are most commonly reported, while Le Fort III fractures are the least frequently described [40,42,44].

Nasal bone fractures have been reported in approximately 7–12% of cases [36,40,42,43,44]. Orbital bone fractures occur in about 1–29% of cases [40,42,43,44], while frontal bone fractures resulting from bicycle accidents are observed in approximately 1–4% of patients [40,42,43,44]. In addition to craniofacial fractures, minor soft tissue trauma such as abrasions, lacerations, or ecchymoses may also occur [42].

Furthermore, the location of fractures may be influenced by the type of accident. In the studied population, maxillofacial injuries were most commonly bilateral; however, in cases where lateralisation was observed, the right side predominated. The dynamics of the accidents may explain this. Among the analysed cases, the most common mechanism of injury, when determinable, was a fall from the bicycle, followed by a collision with a motor vehicle or impact with a pedestrian or obstacle. These findings are consistent with previous studies [36]. In fall-from-bicycle incidents, the rider typically hits the ground sideways, resulting in injuries concentrated on one side of the face. Conversely, in frontal collisions or over-the-handlebar ejections, the injuries tend to be more centrally located [44].

The point of impact significantly affects the fracture pattern. Fractures may occur via a direct mechanism (impact at the site of trauma) or an indirect one, due to force transmission through the bone structures. The anatomical complexity of the craniofacial skeleton facilitates the distribution of forces according to the skeletal buttress system [43], which may explain the observed number of double and multiple mandibular fractures. For instance, impact to the middle or lateral portion of the mandibular body (as in a frontal crash) may result in force transmission leading to condylar fractures [40]. This may also account for the high prevalence of bilateral fractures observed in the study group. Additionally, both anatomical and biomechanical factors explain the significant frequency of midface fractures, particularly involving the zygoma [43]. The zygomaticomaxillary complex is one of the most vulnerable facial areas to external forces, and thus frequently injured in bicycle-related accidents due to impact with obstacles (e.g., vehicle body or ground) [36,40,43].

Bicycle-related accidents may also result in injuries to other body regions. The most common form of craniocerebral trauma accompanying maxillofacial injuries is traumatic brain injury [43,46]. Protective helmets are a fundamental component of cyclist safety gear, and their use reduces the risk of head injuries [47,48,49]. The rate of helmet use among victims sustaining maxillofacial injuries varies across studies, ranging from 3% to 14.8% [42,43,45,50]. Some research does not demonstrate a statistically significant difference in the incidence of injury between helmeted and non-helmeted riders [43]. However, a systematic review and meta-analysis confirmed that helmet wearers sustain significantly fewer maxillofacial injuries than those not wearing helmets [51].

It is important to note that the protective effect of helmets differs depending on the facial region. The lower face area remains particularly vulnerable to injury, even with helmet use [52]. Alcohol consumption is another factor influencing cycling safety, as it impairs the ability to operate a vehicle. Alcohol has been found in the blood of up to 32% of victims with bicycle-related maxillofacial trauma [43,44]. In the present study, alcohol use was associated with specific injury patterns. Some studies also show a statistically significant increase in the number of fracture lines among intoxicated victims [43]. Additionally, our analysis revealed that alcohol-intoxicated cyclists were significantly more likely to experience a fall from the bicycle, potentially due to the substance’s adverse effect on balance control.

Concerning treatment, the majority of patients sustaining bicycle-related maxillofacial injuries required hospitalisation. Moreover, surgical intervention was the predominant treatment approach in most cases involving fractures. The average length of hospital stay was approximately 5 days [43].

### Study Limitations

One of the main limitations of this retrospective study is the lack of detailed information regarding the circumstances of bicycle-related accidents in some cases, including the specific type of accident or helmet use. This omission limits the ability to assess the protective role of helmets directly, and future prospective observational research should incorporate helmet-use data to strengthen such analyses. The limitations of missing data are particularly relevant in cases of patient transfers from other hospitals without maxillofacial surgery capabilities. Furthermore, some relevant cases may have been excluded due to possible omissions in the medical records concerning the exact aetiology of injuries and accident details. Such situations may include patients presenting for diagnostic assessment several days post-incident following an initial consultation in primary care settings. More rigorous and standardised data collection—such as integration with emergency medical service reports or trauma registry logs—should be considered in future registries to reduce the proportion of missing cause-of-injury data.

Additionally, as routine toxicology screening for alcohol or psychoactive substances is not consistently performed in patients requiring medical attention after traffic accidents, determining the exact percentage of individuals under the influence at the time of injury remains limited. Moreover, some patients may seek medical help with a delay, after the alcohol has been eliminated from the organism, which may be due to legal provisions strictly regulating driving under the influence of alcohol. Only five patients tested positive for alcohol, substantially limiting the statistical power to detect meaningful associations between alcohol consumption and injury patterns. Moreover, the lack of precise accident reconstruction data limits the ability to determine the speed at the time of the incident, which may significantly impact the injury outcome.

Further research should also address postoperative outcomes and potential complications in individuals who sustain maxillofacial trauma as a result of bicycle-related accidents. This is particularly important, as fractures in the facial region may lead to functional, aesthetic, or psychological impairments.

## 5. Conclusions

Maxillofacial injuries from bicycle accidents are a significant clinical concern, often involving the lower and midface and frequently requiring hospitalisation and surgery. These injuries may also be linked to life-threatening craniocerebral trauma. Understanding typical injury patterns and risk factors, including the impact of alcohol, is crucial for developing effective prevention, diagnostic, and treatment strategies. The findings also support the need for improved protective measures, such as enhanced helmet designs focusing on lower facial protection. Educational efforts to raise cyclist awareness, especially during peak accident seasons, should be intensified. Finally, further research with larger populations is necessary to explore additional risk factors, including substance use, protective equipment, and environmental conditions.

## Figures and Tables

**Table 1:** Participants characteristics.

	M/*n*	Q1–Q3/%
**Age, years**	38	29–51
**Hospitalisation, days**	6	4–9
**Gender**		
males	70	70.71
females	29	29.29
**Nationality**		
Polish	95	95.96
others	4	4.04

**Table 2:** Frequency of injuries depending on year, month, and day of week.

	*n*	%
**Year**		
2019	19	19.19
2020	29	29.29
2021	29	29.29
2022	8	8.08
2023	14	14.14
**Month**		
I	0	0.00
II	5	5.05
III	6	6.06
IV	7	7.07
V	11	11.11
VI	14	14.14
VII	16	16.16
VIII	14	14.14
IX	15	15.15
X	4	4.04
XI	4	4.04
XII	3	3.03
**Day**		
Monday	12	12.12
Tuesday	13	13.13
Wednesday	11	11.11
Thursday	11	11.11
Friday	20	20.20
Saturday	17	17.17
Sunday	15	15.15

**Table 3:** Detailed characteristics of injuries.

	*n*	%
**Fracture kind**		
ZMO	30	30.30
Orbital	11	11.11
Frontal	10	10.10
Zygomatic	12	12.12
Nasal	3	3.03
Maxilla	8	8.08
LeFort1	5	5.05
LeFort2	9	9.09
LeFort3	1	1.01
Mandible = 1	18	18.18
Mandible = 2	19	19.19
Mandible ≥ 3	7	7.07
Others	3	3.03
**Fracture site**		
left	25	25.25
right	32	32.32
both	42	42.42
**Injury reason**		
undefined	70	70.70
fall	19	19.19
vehicle	4	4.04
pedestrian	3	3.03
barrier	3	3.03
**Treatment method**		
operative	96	96.97
ambulatory	3	3.03
Other craniocerebral injuries	5	5.05
Other consultations	16	16.16

**Table 4:** Comparison of injury characteristics depending on gender, age, and alcohol intake—data presented as percentages.

	Gender			Age			Alcohol		
	Males *n* = 70	Females *n* = 29	*p*-Value	≤30 *n* = 30	>30 *n* = 69	*p*-Value	No *n* = 94	Yes *n* = 5	*p*-Value
**Fracture kind**									
ZMO	30.00	31.03	0.919	23.33	33.33	0.320	29.79	40.00	0.628
Orbital	11.43	10.34	0.876	6.67	13.04	0.354	11.70	0.00	0.417
Frontal	12.86	3.45	0.157	10.00	10.14	0.982	7.45	60.00	<0.001 *
Zygomatic	10.00	17.24	0.315	3.33	15.94	0.077	11.70	20.00	0.580
Nasal	4.29	0.00	0.258	0.00	4.35	0.246	3.19	0.00	0.685
Maxilla	8.57	6.90	0.781	3.33	10.14	0.253	8.51	0.00	0.496
LeFort1	7.14	0.00	0.140	0.00	7.25	0.130	5.32	0.00	0.597
LeFort2	12.86	0.00	0.043 *	6.67	10.14	0.580	7.45	40.00	0.014 *
LeFort3	1.43	0.00	0.518	0.00	1.45	0.508	0.00	20.00	<0.001 *
Mandible = 1	20.00	13.79	0.466	33.33	11.59	0.010 *	18.09	20.00	0.914
Mandible = 2	12.86	34.48	0.013 *	20.00	18.84	0.893	19.15	20.00	0.962
Mandible ≥ 3	8.57	3.45	0.365	16.67	2.90	0.014 *	7.45	0.00	0.527
Others	2.86	3.45	0.876	3.33	2.90	0.908	3.19	0.00	0.685
**Fracture combinations**									
Only mandible	32.86	44.83	0.519	60.00	26.09	0.007 *	38.30	0.00	0.016 *
Only maxillofacial fracture without mandible	37.14	37.93		23.33	43.48		38.30	20.00	
Combined fractures with mandible	8.57	6.90		10.00	7.25		6.38	40.00	
Combined fractures without mandible	21.43	10.34		6.67	23.19		17.02	40.00	
**Fracture site**									
left	22.86	31.03	0.658	30.00	23.19	0.664	26.60	0.00	0.189
right	34.28	27.59		26.67	34.78		32.98	20.00	
both	42.86	41.38		43.33	42.03		40.43	80.00	
**Injury reason**									
undefined	80.00	48.28	0.002 *	70.00	71.01	0.999	72.34	40.00	0.237
fall	12.86	34.48		20.00	18.84		18.09	40.00	
vehicle	2.86	6.90		3.33	4.35		3.19	20.00	
pedestrian	0.00	10.34		3.33	2.90		3.19	0.00	
barrier	4.28	0.00		3.33	2.90		3.19	0.00	
**Treatment method**									
operative	97.14	96.55	0.876	100.00	95.65	0.246	97.87	80.00	0.023 *
ambulatory	2.86	3.45		0.00	4.35		2.13	20.00	
Other craniocerebral injuries	7.14	0.00	0.140	3.33	5.80	0.607	3.19	40.00	<0.001 *
Other consultations	20.00	6.90	0.107	16.67	15.94	0.928	13.83	60.00	0.006 *

* statistical significance.

**Table 5:** Comparisons of hospitalisation duration depending on gender, age, and alcohol intake.

	Gender			Age			Alcohol		
	**Males** ***n* = 70**	**Females** ***n* = 29**	***p*-Value**	**≤30** ***n* = 30**	**>30** ***n* = 69**	***p*-Value**	**No** ***n*** **= 94**	**Yes** ***n* = 5**	***p*-Value**
Hospitalisation, days	6 (4–9)	7 (4–8)	0.657	7 (4–9)	6 (4–8)	0.759	6 (4–8)	11 (10–13)	0.043 *

* statistical significance.

**Table 6:** Multidimensional correspondence analysis—parameters of determined points.

	x	y	z	Quality	Relative Inertia	x		y		z	
						Inertia	cos^2	Inertia	cos^2	Inertia	cos^2
male	−0.260	0.351	−0.396	0.761	0.063	0.034	0.148	0.075	0.269	0.107	0.344
female	0.569	−0.769	0.868	0.761	0.137	0.074	0.148	0.164	0.269	0.236	0.344
≤30 y	−1.009	−0.392	−0.153	0.642	0.130	0.258	0.546	0.048	0.083	0.008	0.013
>30 y	0.542	0.211	0.082	0.642	0.070	0.139	0.546	0.026	0.083	0.004	0.013
mandible	−0.579	−0.526	0.296	0.716	0.099	0.123	0.343	0.124	0.284	0.044	0.090
ZMO	0.697	0.078	−0.942	0.781	0.128	0.128	0.275	0.002	0.003	0.320	0.503
orbital	0.308	1.797	1.440	0.824	0.173	0.009	0.015	0.378	0.493	0.274	0.317
alcohol:0	−0.110	0.088	0.015	0.539	0.007	0.009	0.324	0.007	0.209	0.000	0.006
alcohol:1	2.942	−2.358	−0.390	0.539	0.193	0.227	0.324	0.178	0.209	0.005	0.006

Legend: “alcohol:0”—no alcohol consumption, “alcohol:1”—alcohol consumption.

## Data Availability

Data are available upon request from the corresponding author.
